# Up‐regulated basigin‐2 in microglia induced by hypoxia promotes retinal angiogenesis

**DOI:** 10.1111/jcmm.13256

**Published:** 2017-06-29

**Authors:** Jie Yin, Wen‐Qin Xu, Ming‐Xiang Ye, Yong Zhang, Hai‐Yan Wang, Jian Zhang, Yu Li, Yu‐Sheng Wang

**Affiliations:** ^1^ Department of Ophthalmology Eye Institute of China PLA Xijing Hospital Fourth Military Medical University Xi'an China; ^2^ Department of Ophthalmology Jinling Hospital Nanjing China; ^3^ Department of Pulmonary Medicine Xijing Hospital Fourth Military Medical University Xi'an China; ^4^ Department of Biochemistry and Molecular Biology Fourth Military Medical University Xi'an China; ^5^ State Key Laboratory of Cancer Biology Cell Engineering Research Centre & Department of Cell Biology Fourth Military Medical University Xi'an China

**Keywords:** basigin, microglia, angiogenesis, Retina, hypoxia

## Abstract

Retinal microglia cells contribute to vascular angiogenesis and vasculopathy induced by relative hypoxia. However, its concrete molecular mechanisms in shaping retinal angiogenesis have not been elucidated. Basigin, being involved in tumour neovasculogenesis, is explored to exert positive effects on retinal angiogenesis induced by microglia. Therefore, we set out to investigate the expression of basigin using a well‐characterized mouse model of oxygen‐induced retinopathy, which recapitulated hypoxia‐induced aberrant neovessel growth. Our results elucidate that basigin is overexpressed in microglia, which accumulating in retinal angiogenic sprouts. *In vitro*, conditioned media from microglia BV2 under hypoxia treatment increase migration and tube formation of retinal capillary endothelia cells, compared with media from normoxic condition. The angiogenic capacity of BV2 is inhibited after basigin knockdown by small interfering RNAs. A new molecular mechanism for high angiogenic capacity, whereby microglia cells release basigin *via* up‐regulation of PI3K‐AKT and IGF‐1 pathway to induce angiogenesis is unveiled. Collectively, our results demonstrate that basigin from hypoxic microglia plays a pivotal pro‐angiogenic role, providing new insights into microglia‐promoting retinal angiogenesis.

## Introduction

Retinal pathological neovascularization triggered by hypoxia leads to severe visual impairment and blindness. Retinopathy of prematurity (ROP), especially, occurring in preterm infants, accounts for 6–18% of visual impairment in children 1. Relative hypoxia, following supplemental oxygen therapy required for preterm infant survival, is the crucial cause responsible for abnormal vessel growth in vaso‐obliteration areas in the immature retina and ultimately formation of haemorrhages and retinal detachment 2. Although angiogenesis therapies such as anti‐vascular endothelial growth factor (VEGF) medication with monoclonal antibodies or aptamers have shown promise in clinical studies for adults’ choroidal neovascularization, systemic absorption of those drugs may cause potential side effects on development especially for infants 3. Hence, there is an urgent need to identify alternative therapies addressing the underlying molecular determinants response to hypoxia that stimulates the aberrant vascular growth in ROP.

Microglia cells, the resident macrophages, associates directly with nascent vessels at the vascular front and modulate angiogenesis as key regulators and guides of the forming vasculature 4, 5, 6, 7, 8. Microglia‐deficient mice were found to have reduced numbers of vascular branch points between neighbouring tip cells in the retina 9. The close spatial localization of retinal microglia and blood vessels suggests an emerging role of microglia in vascular angiogenesis and vasculopathy. However, the molecular mechanism underpinning this process has not been fully elucidated.

Basigin, an extracellular matrix metalloproteinase inducer (EMMPRIN, also called CD147), is a pleiotropic transmembrane cell surface glycoprotein. While predominantly recognized for its role in multiple biological processes, such as immune response, tumour progression, matrix proteolysis and cell migration, it has also been shown to be involved especially in neovasculogenesis under hypoxia during tumour invasion and metastasis 10. Especially, it has been reported that basigin may promote angiogenic process not only through its protease‐inducing function by up‐regulating matrix metalloproteinase (MMPs) secretion 11, 12, but also directly by its ability to increase soluble forms of VEGF and VEGFR2 in tumour cells and endothelial cells 13, 14, 15. Recent studies reported that expression of basigin was elevated in monocytes/macrophages and played a role on regulating the inflammatory activities of these cells 16, 17. However, its pro‐angiogenic properties in microglia during retinal angiogenesis have not ever been investigated.

Here, we set out to identify a pivotal effect of basigin in microglia that induces the retinal angiogenic events. Our findings also uncover a new mechanism that basigin mediates retinal neovessel growth by increasing secretion of insulin‐like growth factor‐1(IGF‐1) from microglia under hypoxic condition and therefore promoting tube formation of endothelial cells.

## Materials and methods

### Animal experiments

Animal experimental procedures were performed in accordance with the animal ethics committees of the Forth Military University and also complied with the ARVO Statement for the Use of Animals in Ophthalmic and Vision Research. C57BL/6 mice were housed in a barrier facility with *ad libitum* access to food and water with a 12‐hr light/dark cycle. Oxygen‐induced retinopathy (OIR), a mimic of the retinal angiogenesis in ROP, characterized by a late‐phase of destructive pathological angiogenesis, was induced as previously described 18.At birth, pups and their mothers were randomly assigned to control and OIR groups. Briefly, for hypoxic treatment, P7 pups and their mothers were transferred from room air to an environment of 75 ± 2% oxygen for 5 days and afterwards returned to room air. The oxygen concentration in the chamber was continuously monitored with an oxygen analyser. Under these conditions, abnormal preretinal neovascularization occurred after return to normoxia, peaking at around P17. Age‐matched controls were raised simultaneously in room air from P0 to P17. Totally 48 mice were used in experiments. At indicated time‐points (P12 or P17), mice were killed, and the eyes were enucleated, and the retinas were isolated for immunofluorescence and Western blotting.

### Cell culture

The Rhesus macaque choroid‐retinal endothelial cells (RF/6A) and an immortalized microglial line (BV2) were obtained from the cell bank of the Chinese Academy of Science (Shanghai, China) and routinely cultured in Dulbecco's modified Eagle's medium (DMEM; Invitrogen, Carlsbad, CA, USA), containing 10% foetal bovine serum (FBS; Invitrogen), 100 U/ml penicillin and 100 μg/ml streptomycin in a humidified atmosphere with 5% CO_2_ at 37°C.

### Antibodies and reagents

Microglia were labelled using a rabbit primary antibody targeting ionized calcium binding adapter molecule 1 (IBA‐1) (019‐19741) from Wako (Richmond, VA, USA). Antibodies against basigin (Rabbit polyclonal antibody used for Western blot, ab64616; Rat monoclonal antibody used for immunofluorescence double labelling, ab34016), PECAM (ab9498), IGF‐1 (ab9572), IGF‐1 receptor (ab16890), VEGF (ab51745) and VEGFR‐2 (ab5473) were purchased from Abcam (Cambridge, MA, USA). HIF‐1α (MAB 5382) was obtained from Millipore (Billerica, MA, USA). Antibodies against AKT (9272), P‐AKT (4060), ERK (4695), P‐ERK (4370) and the MEK1/2 inhibitors PD98059, PI3K inhibitor LY294002 were from Cell Signaling (Andover, MA, USA). Antibodies against β‐actin were from Santa Cruz (Santa Cruz, CA, USA). Recombinant human IGF‐1 (100‐11) was from ProteinTech (Rochy Hill, NJ, USA). Secondary antibodies including goat anti‐rabbit conjugated to AlexaFluor 594/CY3 or AlexaFluor 488/FITC were purchased from Beijing ComWin (Beijing, China). SYBR Premix Ex Taq II and Multiscript RT were purchased from TaKaRa (Dalian, China).

### Immunofluorescence procedures

Mice were anaesthetized with sodium pentobarbital and perfused with PBS through the left cardiac ventricle followed by 4% paraformaldehyde. After killng, eyes were enucleated and fixed for 2 hrs (h) by chilled 4% paraformaldehyde in 0.1 M phosphate buffer (PB). Then the anterior segment and vitreous were removed, and the posterior eyecups were cryoprotected in graded sucrose solutions (20%, and 30% in PB). The eyecups were embedded and sectioned vertically at 8 μm with a cryostat maintained at a temperature of −20°C. Sections were used for immunolabelling and stored at −20°C until required. Sagittal cross sections were cooled to room temperature and rinsed three times in PBS. Sections were permeabilized for 10 min., blocked for 30 min., and incubated with primary antibodies overnight at 4°C. Then the sections were incubated for 1 hr with secondary antibody combinations and stained with diamino‐2‐phenyl‐indol (DAPI). Immunofluorescence was visualized using a confocal microscope.

### Western blot analysis

Tissue or cells lysates were prepared in lysis buffer (50 nM Tris‐HCl, 150 nM NaCl, 1% NP40, 0.1% SDS, 0.5% deoxycholate, 1 mM phenylmethanesulfonyl fluoride), and protein concentration was determined by BCA protein assay kit (Sangon Biotech, Shanghai, China). Equal amounts (20 μg) of protein were electrophoresed on a 10% sodium dodecyl sulphate (SDS) polyacrylamide gel and transferred onto a 0.22 mm PVDF membrane from Millipore (Bedford, MA, USA). The primary antibodies were used to probe the membranes at 4°C overnight. The membranes were washed and incubated with secondary antibodies for 30 min. Secondary antibodies were chosen according to the primary antibodies origin. After three washes with TBST, protein band signals were detected with an enhanced chemiluminescence system (Millipore). The density of the band was standardized to that of β‐actin.

### RNA interference

BV2 were seeded at a density of 1.0 × 10^5^/well in six‐well plates and grown to 70–80% confluence before transfection. Transfections were performed with small interfering RNAs (siRNA) or negative controls using Lipofectamine 2000 from Invitrogen according to the manufacturer's instructions. The sequence of synthesized siRNA was listed as follows: Basigin‐2 (BSG2‐317: sense 5′‐3′GGAUCAAGGUCGGAAAGAATT; antisense 3′‐5′UUCUUUCCGACCUUGAUCCTT; BSG2‐458: sense 5′‐3′CCAAUAGCACUGAAGCCAATT antisense 5′‐3′ UUGGCUUCAGUGCUAUUGGTT); the negative control siRNA (sense 5′‐3′UUCUUCGAACGUGUCACGUTT antisense 5′‐3′ ACGUGACACGUUCGGAGAATT). For all experiments, cells were transfected with siRNA 48 hrs before further treatments.

### Quantitative real‐time reverse transcription PCR

Total RNA was isolated by the TRIzol reagent from Invitrogen, and cDNA was synthetized following the manufacturer's instruction (TaKaRa). qRT–PCR was performed in a final volume of 10 μl consisting of 1 μl cDNA, 5 μl SYBR Premix Ex Taq II (Takara), 0.5 μl forward primer, 0.5 μl reverse primer and 3 μl RNase‐free H_2_O (Takara). The specific primers for basigin variants, IGF‐1 and β‐actin were synthetized by Shanghai Sangon Co. Ltd (Shanghai, China), and the sequences of each primer were listed as follows:

IGF‐1 (forward: 5′‐CTTCACACCTCTTCTACCTGGCG‐3′; reverse: 5′‐AAGTAAAAGCCCCTCGGTCCACA‐3′), Basigin‐1 (forward: 5′‐GTGGTGGTTTGAAGGGAATGCTC‐3′; reverse: 5′‐CCGAGTAAGGTGGTTGCGGTCTG ‐3′), Basigin‐ 2 (forward: 5′‐CCGCCCTTCTTATAGAGCCGCAGTGG‐3′; reverse: 5′‐ACAGAGGTTTGGATGGTGCCCGCCGC ‐3′), VEGF (forward: 5′‐AGGGCAGAATCATCACGAAGT‐3′; reverse: 5′‐AGGGTCTCGATTGGATGGCA‐3′), VEGFR‐2 (forward: 5′‐ GGCCCAATAATCAGAGTGGCA‐3′; reverse: 5′‐CCAGTGTCATTTCCGATCACTTT‐3′) and β‐actin (forward: 5′‐GACTACCTCATGAAGATC‐3′; reverse: 5′‐GATCCACATCTGCTGGAA‐3′). The qRT–PCR was run in CFX96 Real‐time system and C1000™ Thermal Cycler (Bio‐Rad, Hercules, CA, USA) according to standard protocol. Briefly, the mixture was denatured at 95°C for 10 sec., annealed at 55°C for 10 sec. and extended at 72°C for 15 sec. for 45 cycles. A melt curve analysis was performed to ensure specific amplification. At least three independent experiments were performed. The results were normalized as the fold change for each mRNA of target gene to β‐Actin, which was calculated from standard curves using the 2^−ΔΔCT^ method.

### Endothelial cell migration assays

All migration assays were assessed in a 24‐well modified Boyden chamber (Transwell chamber, Corning, NY, USA) with the two compartments separated by a polycarbonate filter with an 8‐mm pore size. Briefly, 1 × 10^4^/ml RF/6A were placed in the upper chamber in a final volume of 200 μl of serum‐free medium, and conditioned medium and negative controls diluted in the same medium were added to the bottom chamber for a final volume of 500 μl.

The chambers were incubated at 37°C and 5% CO_2_ for 24 hrs. The cells migrating through the filter was fixed by 4% paraformaldehyde for 10 min., stained with 1% crystal violet in methanol. The non‐migrated cells on the upper surface of the filter were wiped away with a cotton swab, and the membrane was imaged. The number of the cells migrating to the bottom side of the filter was counted and plotted as the mean number of cells migrated in six nonoverlapping fields by three investigators in three independent experiments under a microscope (ECLIPSE Ti‐U, Nikon, Tokyo, Japan).

### *In vitro* endothelial cell tube formation assay

Tube formation was performed with RF/6A cells alone as well as in co‐culture with hypoxic microglia or conditioned media from hypoxic microglia. The culture condition of BV2 for co‐cultures is the same as that for conditioned media, both added 10% serum. *In vitro* endothelial cell network formation was assayed using Matrigel obtained from BD (Bedford, MA, USA). Briefly, each well of 96‐well plates was pre‐coated with 50 ul of Matrigel and incubated at 37°C for 1 hr in a 5% CO_2_ incubator. Microglia were seeded into 6‐well plates and exposed to hypoxia for 24 hrs. The culture media was centrifuged, and the supernatant was saved for stimulation of tube formation or other further experiments. A sum of 1.5 × 10^4^ RF/6A cells were seeded per well on the Matrigel and cultured in different medium for 20 hrs. Tube formation at indicated time‐points was acquired by an inverted microscope. Two random images were captured per well. The length of the tubes was measured using Image Pro Plus software, and the results are expressed as mean fold change of tube length compared with the control group. For each experiment, at least three wells per condition were seeded, and each experiment was replicated three times.

### Conditioned medium collection and processing

Conditioned medium (CM) was obtained from the same number of BV2 cell as was applied in the co‐culture experiment. Cells were maintained in DMEM and incubated under the same standard hypoxia treatment. Then CMs were obtained from culture supernatant by centrifuged at 2000× *g* at 4°C for 10 min. to remove cell debris, filtered with a 0.2‐μm polyethersulfone membrane. The CM was used to treat RF/6A cells that to determine its effects on tube formation. In siRNA silencing experiment, BV2 cells were transfected with basigin‐2 siRNA or mock siRNA for 48 hrs. Then the medium was collected and processed.

### Cytokine quantification

The secretion of soluble pro‐angiogenic factors by BV2 cells was evaluated in duplicate using the mouse antibody array kit (AAM‐CYT‐4, RayBiotech, Norcross, GA, USA). To maximum reduce background signal for cytokine array, CM was generated under FBS‐free condition. Briefly, CM media were obtained after the incubation of 2 × 10^6^cells in 5 ml serum‐free medium for 24 hrs at 37°C and 5% CO2. Total protein was extracted, and the concentrations of proteins were standardized. The signal from the membrane was detected with a chemiluminescence imaging system. The signal intensity was quantified by densitometry. The value altered by twofold or more was statistically significant. A positive control was used to normalize the results from different membranes.

### Statistical analysis

All experiments were performed at least in triplicate and repeated at least three times independently. Data are expressed as mean ± S.D. Student's *t*‐test was performed when two groups were compared. Multiple comparisons referring to more than two groups, a one‐way anova was performed, in conjunction with Student–Newman–Keuls (SNK) test. All statistical analyses were performed using GraphPad Prism 5.0 (GraphPad, La Jolla, CA, USA). A value of *P* < 0.05 was considered statistically significant.

## Results

### Basigin was overexpressed in retinal microglia near angiogenic sprouts in OIR mouse model

**Figure 1:**
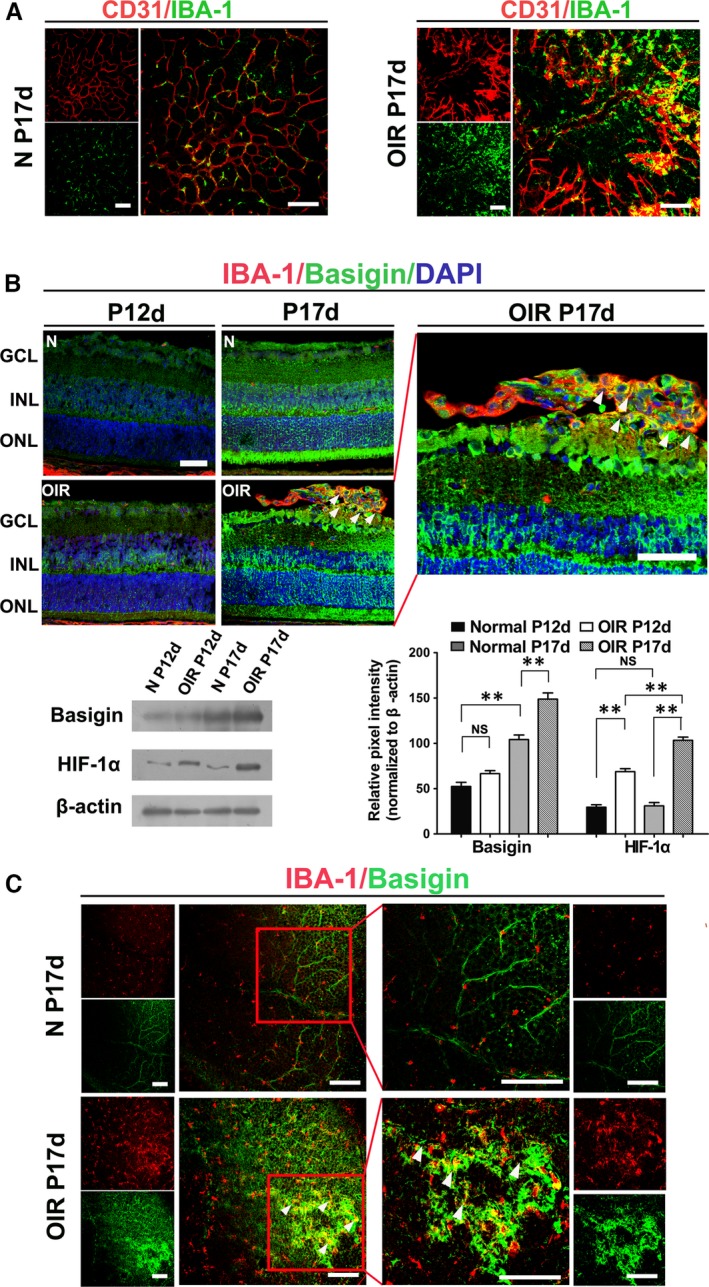
Basigin was overexpressed in microglia, which accumulated in angiogenic sprouts in OIR. (**A**) Confocal images of IBA‐1(microglia marker) and CD31 stained retinal flat mounts in normoxic control mice (N) and OIR mice (OIR). (**B**) Confocal images of IBA‐1 and basigin stained retinal serial cryosections in two groups of mice (N and OIR). White arrowhead points to basigin + microglia associated with preretinal pathological tufts. GCL, ganglion cell layer; INL, inner nuclear layer; ONL, outer nuclear layer. The expression of basigin and HIF‐1α in the neural retina was analysed by Western blot. ** *P* < 0.01; NS no significant difference as determined using one‐way anova. (**C**) Confocal images of IBA‐1 and basigin stained retinal flat mounts at P17 in two groups (N and OIR). High‐magnification images of the boxed areas are shown in the right panels, revealing co‐localization of basigin and IBA1 with vascular front at P17 in OIR mice. White arrows, point to basigin expression positive microglia surrounding angiogenic tufts in the retina of OIR mice. Scale bars: 100 μm (**A** and **C**); 50 μm (**B**).

Firstly, to confirm whether microglia partake in the vascular pathogenesis associated with proliferative retinopathies, we carried out immunofluorescent double staining analysis in retinal flat mounts. OIR mouse model, 75% oxygen from 7th post‐natal day to 12th post‐natal day (P7–P12) to induce vaso‐obliteration and room air until P17 to attain maximal preretinal neovascularization was induced as previously described 18. In contrast with control normal mouse, where a paucity of microglia accumulation within the perivascular niche was observed, our data revealed significantly higher numbers of IBA^+^ microglia localized in close proximity to endothelial sprouting pathological tufts during maximal neovascularization at P17 in the retina (Fig. 1A), which underlying an important functional role for microglia in angiogenesis.

Secondly, we sought to elucidate the expression pattern of basigin in microglia during the progression of disease by immunofluorescent double staining in serial sections. Generally, the intensity of the fluorescence for basigin observed in the nerve fibre layer (NFL) was faint at P12 compared with which observed at P17, just the same with the pattern in previous description about expression levels of basigin in the developing mouse retina^19^, ^20^. At P12, although almost morphologically differentiated, retinal cells are incompletely mature. While faint basigin expression localized at the retinal ganglion neuron layers (GCL), there were general staining of the outer half of the inner nuclear layer (INL), inner segment regions of the photoreceptor cells and blood vessel walls. There was no obvious difference between normal control group and OIR group at P12. A robust increase of immunoreactivity for basigin across the entire neural retina and pigmented epithelium was observed at P17. In normal mice neural retina at P17, basigin staining was identified in the GCL and the INL (Fig. 1B). In the OIR group, basigin also localized to the GCL and, to a lesser extent, to the INL. Notably, the abundant basigin positive IBA^+^ microglia appeared to be co‐localized in the vicinity of or directly associated with neovascular tufts, which clearly outlined by their locations in the vitreal side of the inner limiting membrane into the vitreous in serial sections.

The variation of basigin expression in Western blot was in accordance with variations of immunoreactivity. HIF‐1a expression was also investigated in conjunction with basigin expression in Western blot and the results showed that in normal control mice, the expression was faint all along. In OIR group, the expression slightly increased when finishing 5 days of hyperoxia to room air at P12, peaking at P17, conforming to the conversion of hypoxic situation.

Lastly, immunofluorescence on retinal flat mounts also revealed that basigin^+^ microglia were intimately associated with pathological neovascular tufts at P17 of OIR group (Fig. 1C). Basigin was significantly overexpressed in OIR group mouse retinas at P17 compared with the control mice. White arrows in Figure 1C pointed to basigin^+^ microglia associated with preretinal tufts. Basigin was also expressed by endothelia cell on the endothelium of neovascular tufts as previously reported 14, 15.

### Up‐regulation of basigin‐2 expression in BV2 cells under hypoxic condition

**Figure 2:**
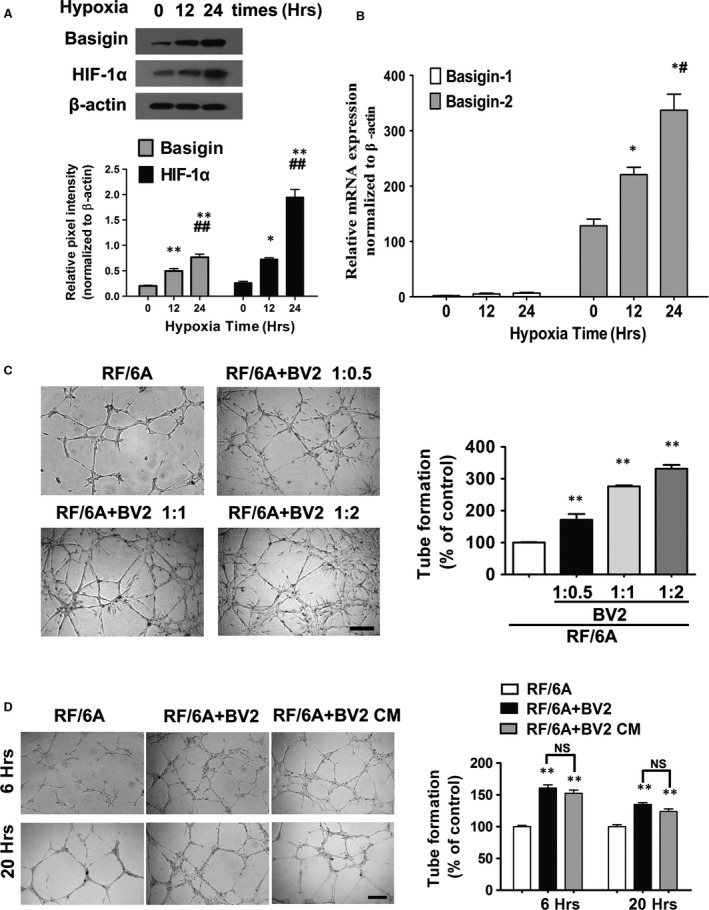
Hypoxia up‐regulate basigin and HIF‐1α a expression in BV2 *in vitro* after 12, 24 hrs of treatment compared with untreated controls. Quantitative analysis of the expression of basigin and HIF‐1α by Western blotting under hypoxia after 12 or 24 hrs, with 0 hr as the baseline (**A**) and real‐time PCR analysis of the expression of basigin isoforms (**B**) in hypoxic BV2 cells (*n* = 3). CT values in real‐time PCR analysis (basigin‐1 30.91 ± 1.80, basigin‐2 19.92 ± 0.76, β‐actin 12.56 ± 0.85) confirmed basigin‐1 expression was low. *P < 0.05 compared with baseline; **P < 0.01 compared with baseline; ^#^ *P* < 0.05 compared with 12 hrs group; ^##^ *P* < 0.01 compared with 12 hrs group. NS, no significant difference during groups. Representative images and quantification of *in vitro* tube formation analysis in Matrigel (**C** and **D**). Quantitative assessment of tube formation was performed as described in the Materials and Methods section. (**C**) BV2 microglia were pre‐treated with 3%O2 for 24 hrs. RF/6A were plated at a density of 1.5 × 10^4^ cells/well in every group, three co‐culture groups were added with BV2 at indicated ratio. Comparison between group differences is all statistically significant (**P < 0.01). Scale bar = 250 μm. (**D**) Microglia under hypoxia treatment facilitated tubulogenesis is not necessarily dependent on direct cell‐to‐cell contact. BV2 were pre‐treated with 3%O_2_ for 24 hrs. 1.5 × 10^4^/well RF/6A were plated. Representative micrographs showing RF/6A tube formation cocultured with BV2 at a riot of 1:0.5 after hypoxia treatment or with culture supernatant from hypoxia BV2. Conditioned medium was obtained from BV2 cell cultures incubated under the same hypoxia treatment and with a similar number of cells. The images were captured at 6 and 20 hrs after plating. **P < 0.01 compared with baseline; NS no significant difference during groups. Scale bar = 500 μm.

We investigated the effects of physical hypoxia on basigin expression in a microglia cell line, BV2 cells. The expression of basigin increased in a time‐dependent manner after hypoxia treatment as shown by Western blotting (Fig. 2A). HIF‐1a levels in BV2 cells were also up‐regulated during hypoxia.

Basigin gene encodes for several products by alternative splicing. As the available commercial basigin antibody could not distinguish isoforms, we examined the expression patterns of two basigin isoforms in hypoxic microglia cell lines by quantitative real‐time PCR. Very low or near undetectable basigin‐1 expression was observed compared with basigin‐2 at all time‐points (2.00 ± 0.556 *versus* 128.13 ± 21.11; 5.03 ± 2.47 *versus* 220.71 ± 22.90; 6.56 ± 2.20 *versus* 336.99 ± 50.29, calculated from standard curves using the 2^−ΔΔCT^ method, at 0, 12, or 24 hrs, respectively). CT values also confirmed basigin‐1 expression was poor (Fig. 2B). Our results revealed basigin‐2 is the predominant basigin isoform expressed by hypoxic microglia. Moreover, the expression of basigin‐2 on mRNA transcription mirrored the time course of the basigin protein expression profile.

### Microglia under hypoxia facilitating tube formation was not necessarily dependent on direct cell‐to‐cell contact

We wonder whether microglia cells mediate angiogenesis by direct interactions with endothelial cell or by secreting soluble factors that enhance endothelial cell migration and tube formation. Firstly, we co‐cultured microglia BV2 with chorioid‐retinal endothelial cell line RF/6A to explore the ability of BV2 inducing tube formation of RF/6A. When the addition ration of BV2 was increasing, tube formation by RF/6A was crucially increased (Fig. 2C). The results showed that interactions between RF/6A and BV2 cells in co‐culture promoted tube formation.

We also tested whether the pro‐angiogenic effects of BV2 were necessarily depending on the contact between microglia and endothelial cells. After the first 6 hrs, tubular‐like structures were gradually formed by RF/6A. Notably, tube formation of CM cultured RF/6A was dramatically increased. Moreover, compared with co‐cultured with BV2 group, no statistically significant less endothelial cell networks formed by CM cultured RF/6A both after 6 or 20 hrs (Fig. 2D). Our findings suggest that soluble factors released from hypoxic microglia may play an important role on hypoxia induced angiogenesis.

### CM from hypoxic BV2 *via* basigin‐2‐knockdown impeded the angiogenic capacity of retinal endothelial cell

**Figure 3:**
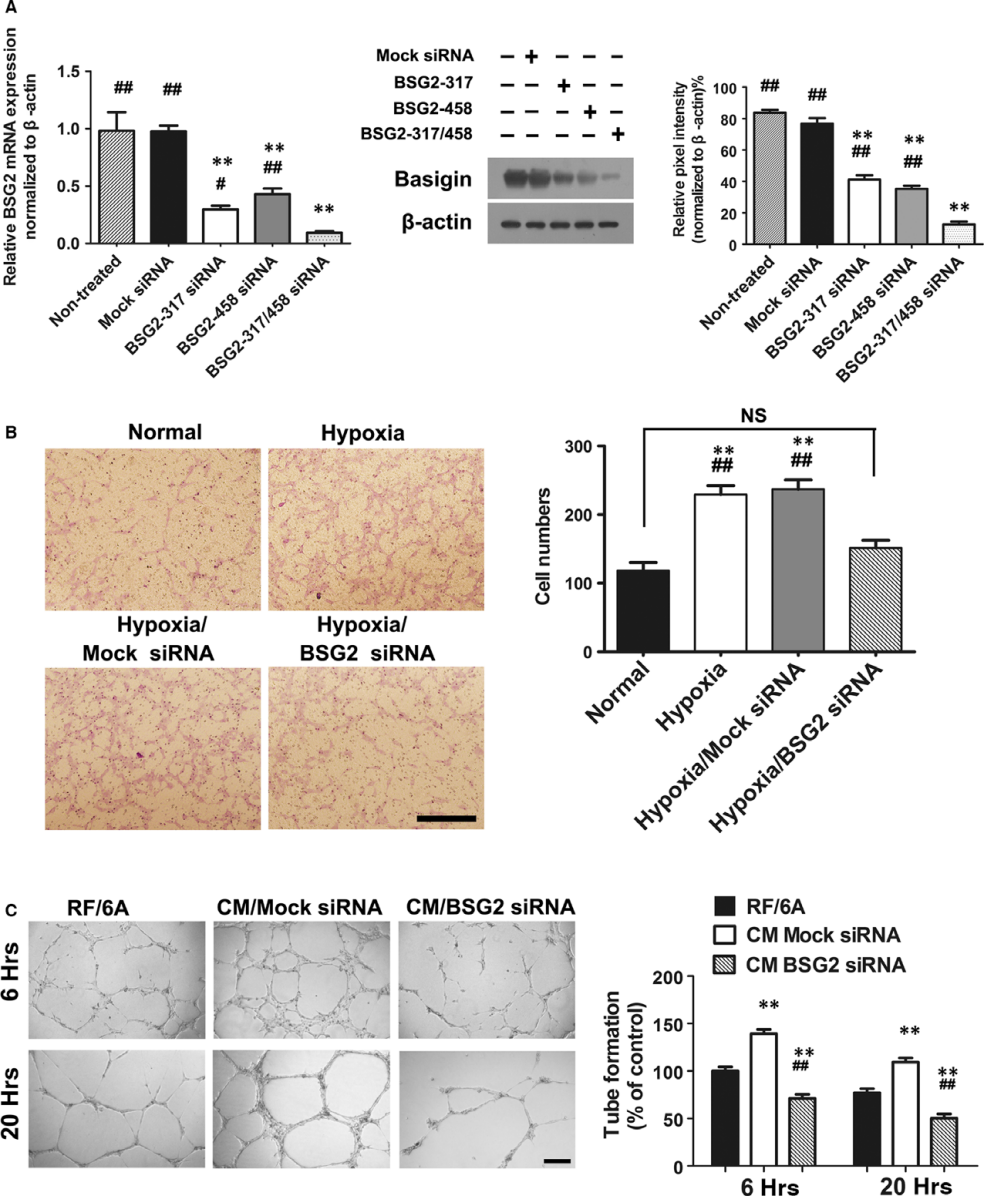
The angiogenic capacity of RF/6A was inhibited when treated with conditioned media from hypoxic BV2 being transfected with basigin‐2 siRNA (BSG2 siRNA). (**A**) Messenger RNA of BSG2 and Protein expression of BSG in BV2 cells transfected with either BSG2 siRNA or Mock siRNA. **P < 0.01 *versus* nontreated group; ^#^ *P* < 0.05 *versus* both siRNA sequence used group; ^##^ *P* < 0.01 *versus* both siRNA sequence used group. (**B**) Transwell migration assay examining the directional migration of RF/6A cells. The cell migration assay was performed as described in the Materials and Methods section. Scale bar = 500 μm. **P < 0.01 *versus* normal group; ^##^ *P* < 0.01 *versus* hypoxia BSG2 siRNA group; NS no significant difference during groups. (**C**) Representative images and quantification of *in vitro* tube formation analysis in Matrigel. The formation of capillary‐like structures was photographed at 6, and 20 hrs. Scale bar = 500 μm. **P < *versus* RF/6A group; ^##^ *P* < 0.01 *versus* CM Mock siRNA group.

Loss‐of‐function experiments were performed with siRNA against basigin‐2, with mock siRNA served as control. To make sure that basigin‐2 was maximally knocked down, we used two different siRNA sequences. The knock‐down effect in BV2 transfected with siRNA was examined by quantitative real‐time PCR and Western blot. Both data at transcription and protein level revealed that the up‐regulation of basigin expression under hypoxia was preserved in the control transfection group, but reduced in the basigin‐2 knocked down group (Fig. 3A). To achieve a higher silence efficiency, when an alliance of two siRNA sequences both at a half dose were simultaneously tried, basigin expression was most profoundly decreased. Thereafter, in later experiments, we used two siRNA sequences combined to interfere with basigin‐2.

To define directly the involvement of basigin from microglia in mediating angiogenesis, we determined RF/6A migration (Fig. 3B), and tube formation (Fig. 3C) in the presence of CM collected from basigin‐silenced BV2. The results showed that the number of RF/6A cells crossing the Matrigel membrane cultured with CM under hypoxia was obviously higher than RF/6A group cultured with normoxia CM (Fig. 3B). However, when RF/6A cells cultured with CM from basigin deficient BV2 cells, the migrating cells were significantly lower than mock siRNA group (*P* < 0.05, Fig. 3B).

When basigin expression was blocked in BV2 cells, tube formations were reduced significantly, compared with untreated controls. SiRNA mediated knock down of basigin led to significantly decreased angiogenesis and reduced the ability of migration in RF/6A (Fig. 3C). All together, these results displayed that tube formation by RF/6A maintained in CM was crucially dependent on basigin expression.

### Microglia‐derived soluble factors, especially IGF‐1, were regulated by basigin‐2

The effects of BV2 supernatant on migration and angiogenesis of RF/6A implicated that microglia‐produced soluble factors act in a paracrine manner. Accordingly, to identify the potential microglia‐derived factors, two paired cytokine antibody arrays were used to examine the expression profiles of several releasable angiogenic factors in supernatant from BV2 under hypoxia or normoxia, transfected by mock siRNA or basigin‐2 siRNA, respectively.

**Figure 4:**
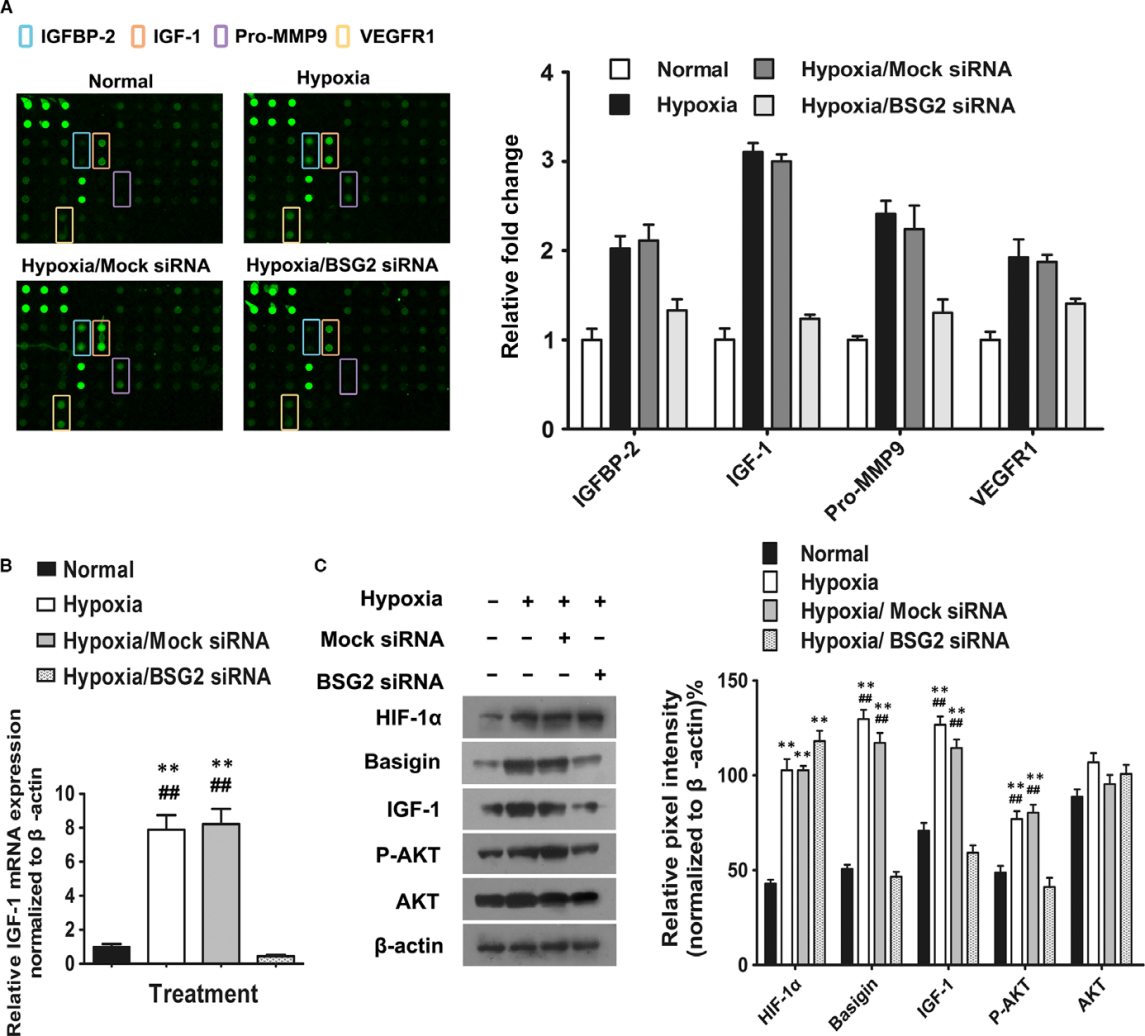
Microglia‐induced endothelial angiogenesis is attributed to Microglia‐derived soluble factors. (**A**) Expression levels of angiogenesis‐related proteins from BV2 in four groups: normal, hypoxia, hypoxia treated with Mock siRNA and hypoxia treated with basigin‐2 siRNA (BSG2 siRNA). Left panel are scanned images of four groups of angiogenesis array data. The first six dots located in upper left corners of the arrays are positive controls and the next four spots are negative controls respectively. Array spots were taken and analysed by Image J software. Expression levels were presented as a mean pixel density normalized by the positive spot references. Right panel showed histogram profile of the relative fold change of four a giogenesis proteins identified from the array study after different treatment. The results were generated by quantifying the mean spot pixel density from the arrays using Image J software. (B) Quantitative real‐time PCR was specifically performed for IGF‐1 after BSG2 siRNA treatment. ** *P* < 0.05 *versus* normal group; ^##^ *P* < 0.05 *versus* hypoxia BSG2 siRNA group. (**C**) Representative Western blot analysis of HIF‐1α, basigin, IGF‐1, AKT and phosphorylated AKT in BV2 cell.

The results showed that four cytokines, including insulin‐like growth factor binding protein (IGFBP)‐2, IGF‐1, Pro‐MMP9 and VEGFR‐1 were up‐regulated under hypoxia but down‐regulated when basigin‐2 was knocked down (Fig. 4A). Among the four identified cytokines, IGF‐1 alteration was most significant. The signals of IGF‐1 in hypoxia medium were increase 3.11‐fold, comparing with that in normoxia medium**,** but decreased to 42.7% (from 3.11‐fold to 1.33‐fold) when knocking down basigin‐2. Moreover, the expression pattern of IGFBP‐2, which may function as a possible additional molecule for IGF‐1 binding and signalling, was similar with IGF‐1. Well‐characterized basigin regulated proteins, such as MMP‐9, was significantly lower in basigin knockdown CM, and was found to be 46.4% of that in the control CM.

Finally, we focused our attention on IGF‐1, the factor modulated maximally by basigin‐2. Consistent findings from BV2 cell extract were obtained. The variations of expression in IGF‐1 after hypoxia treatment and basigin knockdown in BV2 were confirmed at the transcription level (Fig. 4B) and protein level (Fig. 4C).That is, quantitative real‐time PCR showed that hypoxia treatment induced overexpression of IGF‐1 by microglia. Vice versa, the expression of IGF‐1 was markedly decreased when basigin‐2 was knocked down. The expression pattern of IGF‐1 by Western blot agreed with the real‐time PCR results. Moreover, the up‐regulation of AKT phosphorylation was also measured in basigin‐2 deficient BV2. This result indicated that the impeditive effects of basigin‐2 siRNA on IGF‐1 activation might be associated with the inhibition of the phosphorylated AKT signalling pathways.

### Basigin‐2 induced IGF‐1 up‐regulation during enhancement of angiogenesis through AKT phosphorylation activation

**Figure 5:**
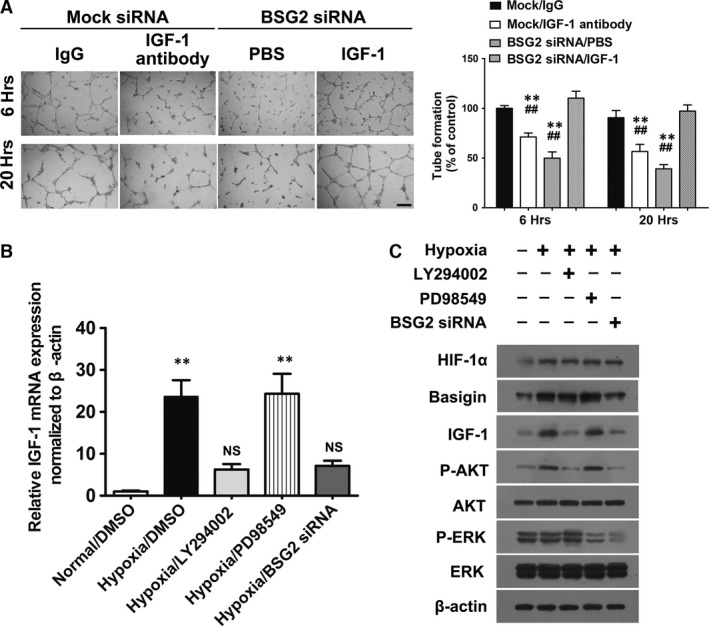
Basigin‐2 (BSG2) induced IGF‐1 up‐regulation during enhancement of angiogenesis through AKT phosphorylation activation. (**A**) Representative micrographs showing RF/6A tube formation stimulated with conditioned media from BV2 transfected with Mock siRNA or BSG2 siRNA. Microphotographs were taken at 6 and 20 hrs after plating. Scale bar = 500 μm. Data represent the mean ± S.D.; **P < 0.01 *versus* IgG group; ^##^ *P* < 0.01 *versus* BSG2 siRNA/IGF‐1 group. (**B**) mRNA expression of IGF‐1 in BV2 treated with different inhibitor or BSG2 siRNA. **P < 0.01 *versus* normal group; NS no significant *versus* normal group. (**C**) Representative Western blot analysis of HIF‐1α, basigin, IGF‐1, phosphorylated AKT, AKT, phosphorylated ERK and ERK in BV2 cell.

We confirmed the role of IGF‐1 on RF/6A treated with neutralized with IGF‐1 antibody. IGF‐1 neutralizing antibody significantly blocked the RF/6A tube formation induced by hypoxic CM as compared with control immunoglobulin G (IgG), Moreover, when IGF‐1 protein was added in CM from basigin‐silenced BV2 cells, tube formations were restored (Fig. 5A).

We subsequently elucidated the possible signalling pathway involved in the enhancement of angiogenesis by basigin‐2. As basigin‐2 knock down by siRNA attenuated AKT activation in microglia (Fig. 4C), we suggested that basigin‐induced IGF‐1 secretion might require AKT. ERK, another key signal molecule proved being regulated by basigin‐2 was also checked.

We found that the down‐regulation of basigin‐2 resulted in the significant decrease of phosphorylated AKT and phosphorylated ERK level, although expression of total AKT and ERK was not affected (Fig. 5C). Remarkably, treatment with a PI3K inhibitor LY294002 or basigin‐2 siRNA both suppressed IGF‐1 expression through inhibition of AKT phosphorylation. However, MEK inhibitor PD98059 did not inhibit IGF‐1 expression though it abolished ERK phosphorylation activation. MEK did not inhibit IGF‐1 expression when ERK activation was abolished. Thus, our data demonstrated that AKT phosphorylation activation was required for induced IGF‐1 up‐regulation by basigin‐2 during enhancement of angiogenesis in BV2 under hypoxia.

### Inhibition of IGF‐1 receptor significantly inhibited tube formation by decreasing VEGFR‐2 expression in RF/6A

**Figure 6:**
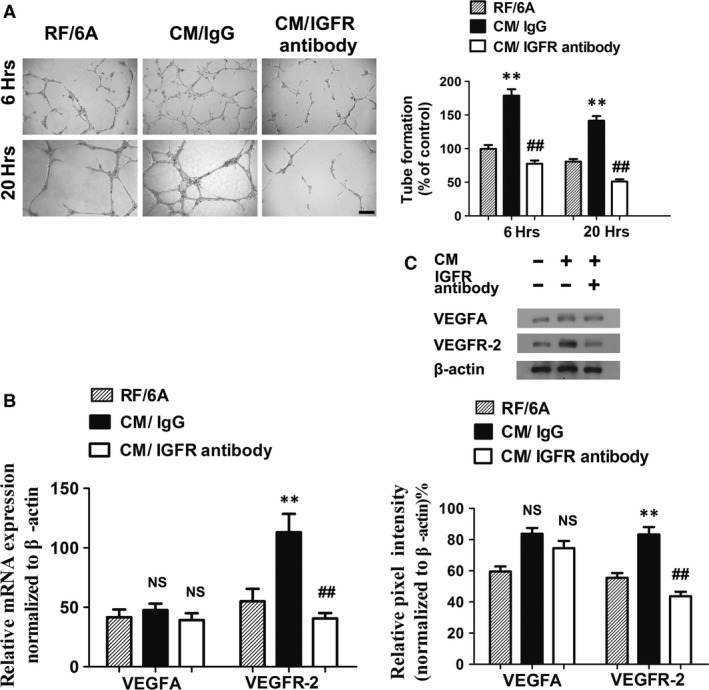
Inhibition of IGF‐1 receptor significantly inhibited tube formation by decreasing VEGFR‐2 expression in RF/6A. **, *P* < 0.01 *versus* RF/6A group; ##, *P* < 0.01 *versus* CM/IgG group; NS, no significant *versus* RF/6A group. (**A**) Representative micrographs and quantification of tube formation of unstimulated RF/6A or RF/6A stimulated with conditioned medium from BV2 in the presence or absence of IGF‐IR (200 ng/ml) treatment. Microphotographs were taken at 6 and 20 hrs after plating. Scale bar = 500 μm. (**B**) Messenger RNA expression of VEGF and VEGFR‐2 in RF/6A with or without IGF‐1 receptor neutralized antibody treatment. (**C**) Western blot analysis of VEGF and VEGFR2 expression in RF/6A under different treatments.

To further underlie the significance of IGF‐1 in new vessel formation, we inhibited IGF‐1 receptor by a neutralization antibody, which led to a significant reduction in tube formation (Fig. 6A). VEGF mRNA expression in RF/6A cells was not affected by IGF‐1 signalling, regardless of CM or IGF‐1 receptor neutralization antibody treatment. In contrast, mRNA expression of VEGFR‐2 was induced by CM, whereas the addition of IGF‐1 receptor neutralization antibody abrogated it (Fig. 6B). In agreement with these findings, Western blot analysis of VEGF protein expression was not affected by the treatment, whereas the expression of VEGFR‐2 protein revealed an IGF‐1 receptor signalling‐dependent manner (Fig. 6B and C).

**Figure 7:**
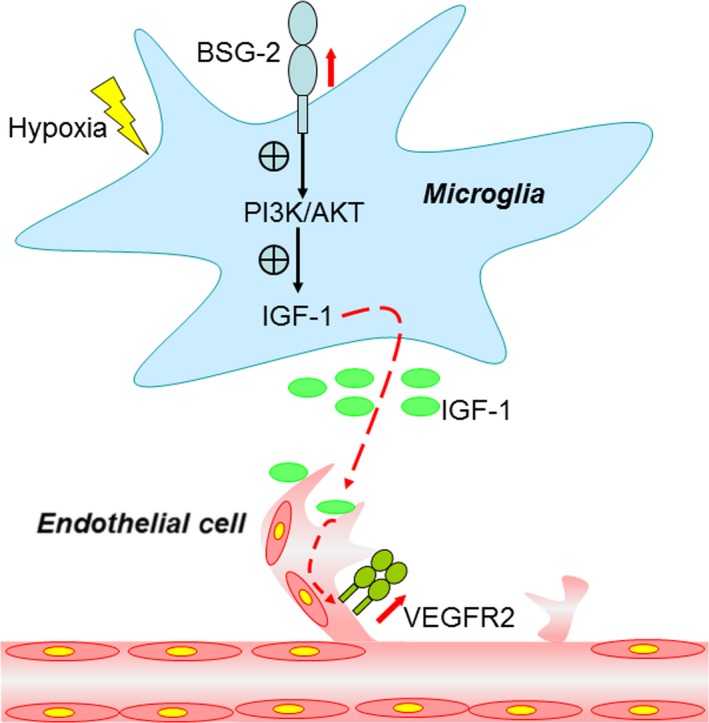
A schematic diagram for microglia‐derived basigin induction and its effect on angiogenesis. The up‐regulated basigin in microglia in response to hypoxia enhances angiogenic capacity of endothelial cells by elevating IGF‐1 *via* PI3K/AKT signaling pathway. BSG‐2, basigin‐2; PI3K, phosphatidylinositol 3‐kinase; AKT, also known as protein kinase B (PKB); IGF‐1, insulin‐like growth factor‐1; VEGFR2, vascular endothelial growth factor receptor 2.

## Discussion

Microglia cells play pivotal roles during vascular development and angiogenesis in the retina and brain 7, 21, 22. Vascularization is preceded by microglia precursors accumulating in the brain 5, 23. Although the contribution of microglia/macrophages to ocular neovascularization has been established for over a decade, knowledge of the mechanisms by which they physically localize closely to areas of angiogenesis and partake in microenvironment interaction remains limited. It is worth noting that microglia, with a highly increased motility, can sense physiological and pathological cues, survey and communicate with the surrounding microenvironment by secreting various cytokines and neurotropic factors 24. Thus, in ROP, microglia can bridge local hypoxia to an innate adaptive response in neovascular retinal disease.

Numerous studies have shown that basigin acts as an angiogenesis enhancer in tumour progression. Recent biochemical analysis indicates putative hypoxia‐responsive elements within its promoter and inhibition of basigin by pharmacological or molecular approaches provokes a therapeutic strategy for cancer 10, 25. Basigin‐2 (NM_198589) is a major isoform of basigin, and it is ubiquitously expressed. While basigin‐1 transcript (NM_001728), with a third immunoglobulin domain (Ig0), is only expressed specifically in the retinal photoreceptors 19, 26, 27. Basigin‐1, associating with a protein secreted from rods, being named as rod‐derived cone viability factor (RdCVF) and glucose transporter (Glut)‐1, form a complex at the cone surface to increase glucose uptake and promotes cone survival 28, 29. Consistent with the tissue specificity of basigin‐1, our results have shown that microglia BV2 cells selectively express basigin‐2. Due to the special localization of basigin‐1 in retinal photoreceptors, it does not directly be involved in the retinal microglial angiogenesis. We prominently focus on basigin‐2 in the following experiment.

To explore whether the angiogenetic activity of microglia depends on its direct contact with retinal endothelial cells or paracrine signalling, two paradigms of culture systems are utilized. Fantin's study elaborately revealed that microglia was in contact with neighbouring endothelial cells and concluded microglia bridging vascular sprouts 21. We also notice microglia BV2 cells residing at the branch point of the tubular‐like structures *in vitro* and in close proximity to endothelial tip cells of the growing vascular tree *in vivo*. However, the angiogenetic efficacy is equally sufficient in cell contact‐free CM culture system. Confirming and extending previous results 9, our data reveal that soluble factors released by microglia mediate angiogenesis and IGF‐1 presents as the leading functional cytokine.

Plenty of studies have shown IGF‐1 induce angiogenesis and endothelial proliferation both in zebrafish cardiovascular and human lung development 30, 31. Notably, previous studies have implicated that IGF‐1 is up‐regulated by microglia/macrophages after hypoxic‐ischaemic injury and contribute to proliferation of microglia 32, 33. Similarly, our work has confirmed that microglia participate in hypoxia triggered angiogenic events by transferring a series of pro‐angiogenic factors, including IGF‐1 and MMP‐9, which mediate various steps of angiogenesis. Angiogenesis also requires the activation of VEGF‐VEGFR signalling. Indeed, endothelial cells engaged in angiogenesis express numerous VEGFRs, but they produce only low levels of endogenous VEGF themselves 34. This might be a rational explanation of the finding that VEGF expression alterations are not in accordance with VEGFR‐2 in RF/6A cells. VEGFR‐2 is the main receptor VEGF exerts its angiogenic effects 35, 36, 37. Our results reveal that IGF‐1, being secreted by hypoxic BV2 to the local microenvironment, stimulates VEGFR‐2 transcription and expression in RF/6A and induces the resultant angiogenesis. Macrophage cells may influence neovascularization indirectly by stimulating VEGF production by neural or glial sources or by producing pro‐angiogenic factors different from VEGF during tumour vascularization 38, 39. Recent studies have shown that abundant VEGF derived from retinal ganglion cells 40, 41, 42, Müller cells 43, 44, 45 and RPE 46, 47 can stimulate endothelial cells and the resultant promotion of retinal angiogenesis in the OIR model, diabetes mellitus model or experimental choroidal neovascularization, respectively.

Studies have shown that low serum IGF‐1 is associated with development of severe ROP 48, 49, 50. IGF‐1 supplementation may be a treatment option to prevent the occlusion of vasculature during the first phase of ROP. However, initiating IGF‐1 supplementation for those developed ROP in vasoproliferative stage would be harmful 3, 51. IGF‐1 has high binding affinity for IGFBP‐2, and this interaction is thought to play a significant role in modulating IGF‐1 localization to its receptor, protecting it from degradation, facilitating its transportation to the injury site. Although, cellular origins of IGFBP‐2 and mechanistically an IGF‐1‐dependent or independent pathway were still in doubt, previous reports have shown IGFBP‐2 increased after hypoxic‐ischaemic injury to the brain 33, and co‐localized with endogenous IGF‐1 at the injury site 32, 52.

Hypoxia is a major molecular controller of angiogenic switch. Especially in retina, hypoxia or ischaemia is the key contributor to a serial of debilitating retinal diseases, like ROP, diabetic retinopathy (DR) and retinal vein occlusions (RVO), which are all characterized by vasoproliferative pathological angiogenesis. Although it is well known that HIF‐1a, the key to mediating hypoxia‐responsive genes, is a major regulator of VEGF, recent *in vitro* studies reveal that additional growth factors, such as IGF‐1, are involved in HIF‐1a expression and subsequently inducing maximally VEGF expression^53^, ^54^, ^55^. IGF‐1 induced VEGF expression in colon and prostate cancer directly and/or indirectly mediated by HIF‐1a 56, 57, 58. What is worth noting, previous reports revealed that there existed a positive feedback mechanism between IGF‐1 and HIF‐1 regulating the interaction of tumour and HUVECs and promoting angiogenesis 56, 59, 60. Our results demonstrate that basigin‐2 could positively regulate the secretion of IGF‐1 from microglia. These data agree with previous report describing existence of positive feedback between basigin‐2 and IGF‐1 in human tumour cells during angiogenesis 61. Therefore, it is entirely possible that the initial activation of HIF‐1 induce basigin‐2 and IGF‐1 up‐regulation. Those angiogenic mediators interact and constitute a positive autocrine loop to maintain pro‐angiogenesis microenvironment.

With respect to the cellular location of basigin, in line with previous studies, we find that basigin also expressed abundantly in vascular endothelial cells 14, 15. The synchronistic localization in endothelial cells and microglia suggests key functional roles of basigin in reciprocal interactions between these two cells to facilitate angiogenesis. However, to obtain insights into whether or how basigin in endothelial cells acts on microglia, further investigations are awaited in future.

Collectively, we first demonstrate the pro‐angiogenic role of basigin‐2 through microglia‐endothelial communication during ROP. Basigin‐2 from hypoxic microglia may enhance endothelial cells cord formation through increasing IGF‐1 secretion. Our study provides the rationale for the therapeutic trials of blocking basigin‐induced angiogenesis for the treatment of hypoxia and ischaemic induced retinal angiogenesis.

## Conflict of interest

The authors confirm that there are no conflicts of interest.
